# The Role and Progress of Antimicrobial Peptides in Managing Oral Biofilms: A Narrative Review

**DOI:** 10.1016/j.identj.2025.104022

**Published:** 2025-11-13

**Authors:** Deji Ciren, Xiaoyue Xia, Yuemeng Zhu, Sumin Hui, Lihua Hong

**Affiliations:** a School and Hospital of Stomatology, Jilin University, Changchun, China; b Department of Second Outpatient Clinic, Hospital of Stomatology, Jilin University, Changchun, China

**Keywords:** Antimicrobial peptide, Plaque biofilm, Bacteria, Oral disease

## Abstract

Oral chronic infectious diseases are intrinsically linked to the formation of dental plaque biofilms, which significantly enhance bacterial resistance to antimicrobial agents and host defenses. The widespread misuse of antibiotics has further compounded this issue by selecting for drug-resistant microbial populations, complicating the clinical management of biofilm-associated oral diseases. Antimicrobial peptides (AMPs) have emerged as promising therapeutic agents due to their excellent biocompatibility, broad-spectrum antimicrobial activity, and low propensity for inducing bacterial resistance. This review aims to synthesise the role and latest progress of AMPs in combating oral biofilms, with a specific focus on their application potential against Cariogenic Biofilms, Periodontal Disease Biofilms, Root Canal Infection Biofilms, Peri-implantitis Biofilms, and Candida-associated biofilms. Furthermore, it summarises contemporary knowledge on AMP types, their mechanisms of biofilm inhibition, and advanced strategies for peptide modification and design, thereby bridging a critical gap in the current literature and providing essential insights for future AMP engineering and clinical translation.

## Graphical abstract

**Figure:**
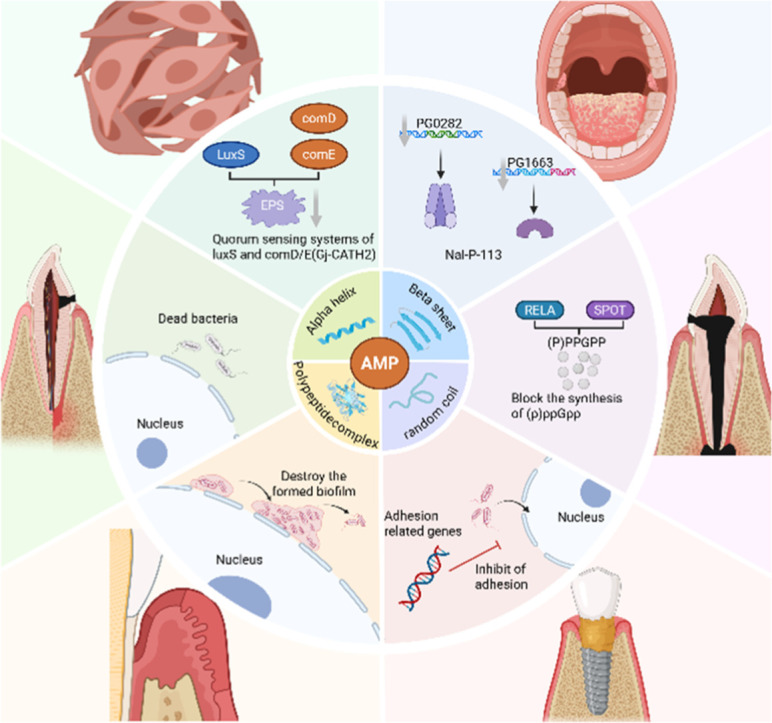

## Introduction

Antimicrobial peptides (AMPs) are expressed by most living organisms, widely exist in various species, including microorganisms, animals, plants and even marine organisms. They play essential roles in defending against bacterial, viral, and fungal infections, as well as adaptive immunity during cancer and autoimmune disease development. Nevertheless, natural AMPs are often subjected to limitations such as weak biological activity, greater toxicity and poor stabilization, which significantly inhibit their applications. To overcome these limitations, rational structural optimization and engineering strategies have been employed.8 These include amino acid substitution, peptide cyclization, sequence truncation, and hybrid design to improve proteolytic resistance and stability. Several AMP-based drugs have become commercially available, with topical and systematic formulations demonstrating safety, efficacy, and low resistance development in clinical use. This narrative review summarises the applications of AMPs in oral diseases such as dental caries, apical periodontitis, periodontal disease, mucosal diseases and oral cancer.

## Results and discussion

AMPs are considered a novel strategy against oral biofilm-related diseases, showing significant antibiofilm effects in experimental studies. Their theoretical and laboratory demonstrated potential often surpasses traditional mouthwashes and natural products like propolis. However, translational challenges remain, including high production costs, stringent regulatory approval, and long-term safety assurance, which must be addressed before widespread clinical application.

The human oral microbiota encompasses up to 700 microbial species, with approximately 250 species typically colonising individual hosts.^1^ The oral cavity establishes an ideal habitat for microbial proliferation through its humid, thermally stable, and nutrient-rich environment. Consequently, oral biofilms or dental plaque represents a quintessential example of clinical conditions caused by biofilms, universally present in mammalian oral ecosystems. These highly structured bacterial assemblies serve as primary etiological agents for diverse oral infections, including gingivitis, dental caries, periapical periodontitis, periodontitis, and peri-implantitis.^2^ Established therapeutic modalities - such as dental restorations, non-surgical or surgical periodontal treatments, root canal therapy, and dental implants - constitute conventional approaches for oral biofilm eradication. Nevertheless, these interventions frequently exhibit incomplete efficacy against secondary biofilm infections.

Antimicrobial peptides (AMPs) constitute essential components of innate immunological defenses against pathogens across most organisms.^3^ Predominantly cationic and amphipathic, AMPs demonstrate multifaceted biological functions beyond antimicrobial activity, including immunomodulatory, anticancer, and antibiofilm properties.^4^ Primary structural characteristics feature amino acid sequences comprising 5 to over 100 residues (primarily L-configured) with molecular weights of 1-5 kDa.^5^ Secondary structural diversity categorises AMPs into subclasses such as α-helical, β-sheet, mixed α-helical/β-sheet, cyclic peptides, and extended conformations.^6^ Both natural and synthetic AMPs represent promising candidates for developing innovative antimicrobial therapies in oral healthcare.

### Natural AMPs

Natural AMPs in the oral cavity primarily include defensins, cathelicidins, and histatins, which exhibit potent antimicrobial activity against oral pathogens and biofilms.^7^^,^^8^

### Defensins

Most defensins adopt β-sheet conformations and comprise 2 peptide families: α-defensins and β-defensins. α-defensins, also termed human neutrophil peptides (HNP-1-4), are stored in azurophilic granules. Lundy et al. documented their robust antibacterial efficacy against S. mutans, S. aureus, E. faecalis, and C. albicans via radial diffusion assays.^9^ Human β-defensins (HBDs), constitutively expressed by epithelial and immune cells, demonstrate therapeutic potential for oral diseases. Salivary HBDs exhibit antibacterial effects against both planktonic bacteria and tooth-adherent biofilms. Within gingival tissues and crevicular fluid, HBDs suppress pathogens and biofilms associated with periodontitis and peri-implantitis. Additionally, HBDs highly expressed in hyperkeratinised epithelium confer protection against oral epithelium infections.^10^

### Cathelicidin

The sole human cathelicidin family member, LL-37, is cleaved from human cathelicidin (hCAP-18) and exerts direct antimicrobial activity by disrupting microbial surface structures. In healthy individuals, LL-37 is expressed in oral epithelium, tonsils, and salivary glands.^11^ Clinically, reduced LL-37 levels correlate with caries susceptibility in patients and children exhibiting high caries activity. Conversely, cathelicidin expression is upregulated in chronic sialadenitis and inflamed gingival tissues.^12^

### Histatins

Histatins are low-molecular-weight cationic salivary peptides synthesised by parotid and submandibular ductal cells in healthy adults. Comprising 3 principal isoforms (His-1, His-3, His-5), they exhibit renowned antifungal and antibacterial activity, particularly against C. albicans.^13^ J. A. R. Curvelo et al. substantiated that His-5 significantly reduces biofilm thickness, inhibits metabolic activity, and demonstrates cytotoxicity against fluconazole-resistant C. albicans biofilms in vitro.^14^

Despite extensive validation of their bactericidal efficacy in vitro, clinical translation of natural AMPs faces significant hurdles. Key limitations encompass restricted natural sources, suboptimal potency, high production costs due to lengthy peptide sequences, susceptibility to proteolytic degradation and high-salt environments, and potential hemolytic/cytotoxic effects.

### Synthetic AMPs

To circumvent limitations of natural AMPs - including short half-life, extended sequence length, and structural instability - researchers employ diverse strategies to design, modify, and synthesise novel engineered AMPs using natural peptide templates. These synthetic analogs exhibit enhanced potency, reduced toxicity, and improved stability.

### Template-based modification design

The key approaches and examples of the template-based modification design strategy for synthetic AMPs are summarized in Table 1. Primary structural optimization through residue addition, deletion, or substitution represents the most prevalent approach for peptide engineering.

**Table 1:** Synthetic AMPs designed based on natural AMP as templates

AMP Source	Fragment Name/Sequence	Modification Method	Function	Reference
Histatins5	Dhvar4 KRLFKKLLFSLRKY-NH_2_	Amino acid substitution KRLFKKLLFSLRKY-NH_2_ KRLFKKLLFWLRKY-NH_2_	Enhanced antibacterial activity of S. mutans	^15^
Cathelicidin LI-37	KR-12 KRIVQRWKDFLR	Amino acid addition KRIVQRIKDFLR KRIVQRWKDFLRKAEK-NH₂	Enhanced antibacterial and antibiofilm activity against S.mutans, broadened antibacterial spectrum	^17^
Human chromogranin (CGA)	ARILSILR HQ	N-terminal fatty acid conjugation RILSILRHQ N-octanoic-RILSILRHQ	Enhanced anti-Candida activity while improving stability	^27^
Histatins5	P-113 AKRHHGYKRKFH-NH₂	Non-natural amino acid substitution AKRHHGYKRKFH-NH Ac-AKRNa1NalGYKRKFNal-NH Ac-AKRBipBipGYKRFBip-NH_2_ Ac-AKRDipDipGYKRKFDip-NH_2_	Enhanced salt tolerance, stability against serum proteolysis	^29^
Cathelicidin LL-37	LL-31 LLGDFFRKSKEKIGKEFKRIVQRIKDFLRNL	D-amino acid substitution	Resistance to gingipain activity	^28^
Cathelicidin LL-37	KRIVQRIKDFLR	Increased peptide chain length KRIVQRIKDFLR KRIVQRWKDFLRKAEK-NH_2_	Addition of KAEK allows better matching of the peptide to membrane thickness, facilitating pore formation through peptide chain binding	^17^
Porcine host defensin	RGGRLCYCRRRFCVCVGR	Protein fusion	Extended half-life of polypeptide drugs	^30^

### Enhancement of antimicrobial activity

Rational AMP design prioritises 5 critical parameters: net charge, hydrophobicity, amphipathicity, secondary structure, and chain length.

### Design based on net charge, hydrophobicity, and amphipathicity

**Fig. 1:**
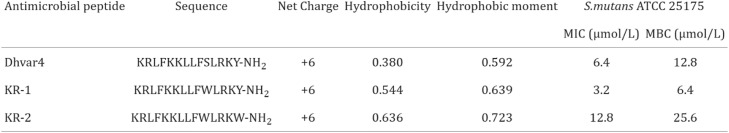
Physicochemical properties and antibacterial activity of Dhvar4 and its derivative peptides.Adapted with permission from Ref.^15^

He et al. engineered KR-1, a short-chain AMP derived from Dhvar4 (itself based on natural His-5), by substituting charged and weakly hydrophobic residues on the nonpolar face with tryptophan. This yielded a rapidly bactericidal peptide with high activity and low toxicity. Enhanced hydrophobicity from tryptophan significantly increased anti-S. mutans efficacy. At 0.6 × MIC, KR-1 reduced S. mutans biofilm formation to approximately 11% (Figure 1).^15^ Notably, KR-2 - the most hydrophobic variant - exhibited the highest MIC, indicating diminished antimicrobial activity when hydrophobicity exceeds a critical threshold. This threshold likely aligns with natural AMPs containing 40-60% hydrophobic residues.

KR-12 corresponds to residues 18-29 of human cathelicidin LL-37.^16^ To enhance its anti-S. mutans and antibiofilm activity, researchers appended KAEK to KR-12′s C-terminus. The 2 lysines increased net positive charge, while the 4 amino acids enhanced amphipathicity. Consequently, the modified peptide demonstrated significantly elevated antimicrobial activity and expanded efficacy against previously insensitive bacteria,^17^ confirming that AMP amphipathicity critically influences antimicrobial spectrum selectivity.

### Secondary structure-based design

Amino acid secondary structural propensity critically determines AMP activity. While disordered in aqueous solutions, most AMPs adopt α-helical or β-sheet conformations (including antiparallel β-strands) in membrane-mimetic environments. For example, human cathelicidin LL-37 transitions from random coil conformation in pure water to stable α-helical structures in saline solutions or varying pH, with helicity directly correlating with antimicrobial potency. Structure-function analysis of bifunctional peptides with 2 different antimicrobial peptide domains revealed TiBP-AMPA exhibits stronger antibacterial activity than TiBP-Gl13K. The AMPA domain contains 2 α-helical motifs versus Gl13K's single α-helix. Higher secondary structure retention in AMPA suggests TiBP-Gl13K holds greater therapeutic potential for peri-implant diseases. Crucially, membrane-bound secondary structural stability drives AMP activity.^18^

### Chain length design

Short-chain AMPs offer advantages in synthetic efficiency, ease of modification, and functional predictability. However, amphipathic structures minimally require 7-8 residues. Within the barrel-stave model, α-helical AMPs necessitate at least 22 residues to traverse bacterial lipid bilayers, while β-sheet AMPs require at least 8 residues. Furthermore, propensity for antimicrobial helix formation (e.g., α-helix/β-sheet) diminishes with decreasing peptide length, with >80% helical content requisite for significant antimicrobial activity. KR-12 exemplifies the known peptide with the lowest natural activity. As previously noted, C-terminal fusion of KAEK enhanced its antibacterial and antibiofilm efficacy. The possible reason is that KR-12′s 12-residue helical structure is insufficient for membrane penetration. The expansion to 16 residues via KAEK provides 4 additional residues (a potential α-helical turn), facilitating membrane thickness matching and pore formation through peptide linkage.^17^ Thus, beyond charge and hydrophobicity, peptide chain length substantially influences antimicrobial efficacy.

Additionally, non-natural modifications such as lipidation - where hydrophilic peptides are covalently conjugated to hydrophobic fatty acids via amide bonds - enable partial insertion of lipid tails into bacterial membranes. This process promotes secondary structure formation in the resulting lipopeptide, thereby enhancing hydrophobicity and membrane-lytic capacity.^19^

### Stability enhancement

#### C-terminal amidation

C-terminal amidation converts carboxylic acid groups (-COOH) to carboxamide groups (-CONH_2_), altering terminal chemistry to impede recognition and cleavage by carboxypeptidases. Natural C-terminally amidated peptides demonstrate enhanced helical stability and lytic activity, with glycine predominantly occupying the C-terminal position.^20^

#### N-terminal acetylation

The engineered antimicrobial peptide L163 exhibits activity against multidrug-resistant (MDR) bacteria^21^ but undergoes rapid degradation in plasma and trypsin. N-terminal acetylation significantly enhances its stability against pH variations, plasma components, and tryptic degradation while concurrently improving antimicrobial efficacy. This protective effect arises from acetylation-mediated blockade of protease-vulnerable sites.^22^ Notably, no studies have reported successful application of N-terminally acetylated AMPs against oral biofilms to date.

#### Peptide cyclization

Cyclization reduces conformational flexibility and enhances proteolytic stability of AMPs. Brevinin-1BW (FLPLLAGLAASFLPTIFCKISRKC), a novel AMP isolated from Pelophylax nigromaculatus skin, exhibits potent activity against clinically resistant S. aureus and E. faecalis. This peptide features a characteristic "Rana box" motif at its C-terminus, formed by an intramolecular disulfide bond between 2 Cys residues. This cyclic structure confers enhanced resistance to proteases (e.g., carboxypeptidases) and stabilization of α-helical conformation, thereby improving both antimicrobial activity and proteolytic resistance.^23^ Consequently, it is evident that post-synthetic modification, specifically the introduction of Cys residues into the sequence, can facilitate the formation of disulfide bonds to create peptide dimers or cyclic structures. This modification guides and stabilises the supramolecular properties of AMPs, which in turn can contribute to their antimicrobial potency. Common cyclization strategies for peptide therapeutics include 4 primary types: head-to-tail, head-to-side chain, side chain-to-tail, and side chain-to-side chain cyclization.^24^

#### Fatty acid conjugation

Fatty acid conjugation may block protease binding sites, thereby improving the proteolytic stability of peptides against proteases.^25^ For instance, CGA-N9, an N-terminal derivative derived from human chromogranin A (CGA),^26^ demonstrated significantly enhanced stability against protease hydrolysis in serum, along with stronger biofilm inhibitory and eradicative capabilities, following N-terminal fatty acid modification by Li et al.^27^

#### D-amino acid substitution

Most AMPs consist of L-amino acids, making them susceptible to proteolytic degradation with consequent short half-lives and compromised stability. D-amino acid substitution decreases proteolytic sensitivity. For example, LL-31, a truncated variant of LL-37, was evaluated alongside its D-enantiomer (D-LL-31) for their antibacterial efficacy within salivary-derived multi-species biofilms. D-LL-31 exhibited superior antimicrobial activity compared to LL-31 in this multi-species biofilm environment. Given that multi-species biofilms possess higher protease content or activity than single-species planktonic cultures, this enhanced activity is reasonably attributed to the differential proteolytic sensitivity of the peptides. The presence of D-amino acids significantly enhances peptide resistance to proteolysis.^28^

#### Non-natural amino acid substitution

Substitution with non-natural amino acids can confer resistance to degradation by disrupting enzyme-substrate binding sites. Researchers synthesised the analogs Bip-P-113 and Dip-P-113 by replacing histidine residues (His4, His5, and His12) in P-113 with β-(4,4′-biphenyl)alanine (Bip) and β-diphenylalanine (Dip), respectively. This modification successfully enhanced the AMPs' antifungal activity against C. species and their stability against serum proteolysis.^29^

#### Protein fusion

Protein fusion refers to a genetic engineering technique where proteins or peptide molecules are fused to functional proteins, creating novel chimeric entities. It is an effective strategy for extending the half-life of peptide therapeutics. Plant-modified antimicrobial peptides (PMAMPs) exhibit a novel capacity for drug delivery to periodontal and gingival cells, demonstrating 13- to 48-fold higher efficiency than other tested cell-penetrating peptides. Therefore, protein drugs incorporating protegrin expressed in plant cells may exert dual functions when delivered as topical oral formulations or chewing gum: delivering therapeutic proteins to gingival tissues while simultaneously killing pathogenic bacteria.^30^

Additionally, the primary cause of cytotoxicity is that during their action, AMPs cannot specifically distinguish between bacterial cell membranes and host cell membranes in vivo, thus nonspecifically lysing cells and causing toxicity. GHaR is a peptide derived from temporin-GHa, possessing significant antimicrobial activity but strong hemolytic toxicity. Therefore, through amino acid substitutions to reduce surface hydrophobicity, GHaR6R, GHaR8R, and GHaR9R were obtained, which not only reduced their cytotoxicity but also demonstrated good antimicrobial activity and antibiofilm activity. Consequently, they show great promise as novel drugs for the prevention and treatment of dental caries.^31^

### De novo design

Based on the α-helical sequence (XXYY)_n_ (where X denotes hydrophobic residues, Y hydrophilic residues, and n repeat count), residues were restricted to basic histidine (His, H) and hydrophobic leucine (Leu, L) to generate amphipathic α-helices, with length constrained to 8-16 residues to minimize helix size. Glycine (Gly, G) was incorporated at position 1 as an N-capping residue. Tryptophan (Trp, W) was positioned at residue 4- the amphipathic interface between hydrophilic and hydrophobic faces - to anchor the peptide to lipid bilayers. C-terminal amidation enhanced net positive charge and antimicrobial activity, yielding novel AMP GH12 (GLLWHLLHHLLH-NH_2_).^32^

### Hybrid peptides

Hybridization of targeting peptides with potent antimicrobial peptides represents a common design strategy. For example, the specifically targeted antimicrobial peptide (STAMP) C16G2 was engineered to target cariogenic S. mutans. It comprises 3 functional domains: C16 (targeting domain, truncated competence-stimulating peptide (CSP) pheromone) accumulates on S. mutans surfaces; G2 (killing domain, truncated Novispirin G10); linked by a flexible triglycine spacer.^33^

### Self-assembling AMP-based nanoparticles

**Fig. 2:**
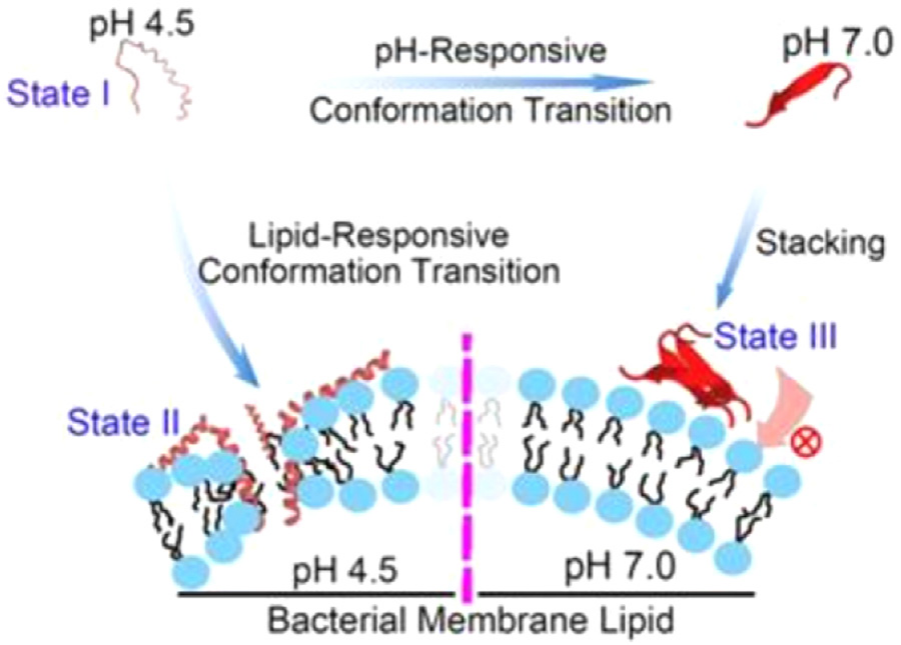
Schematic illustration of the pH- and lipid-responsive conformational transition of peptide pHly-1 and its antibacterial activity associated with the states sensitive to the acidic condition and lipid bilayer penetration. Adapted with permission from Ref.^34^

Dual-sensitive antimicrobial peptide nanoparticles (pHly-1 NPs) were derived from cytolysin-I via lysine substitution, histidine addition, charge modulation, and hydrophobicity optimization. In acidic cariogenic biofilms, protonation of imidazole groups from 7 histidine residues (pKa ≈ 6.0) induces a random coil conformation in pHly-1. Electrostatic repulsion between peptides drives formation of small nanoparticles (State I). Subsequent bacterial membrane binding triggers a conformational shift to amphipathic α-helical structure, enabling membrane penetration and bactericidal activity (State II). Conversely, under normal physiological conditions, imidazole groups in the residues are often neutral, which promotes intermolecular hydrogen bonding, resulting in β-sheet conformation. These β-sheets assemble into nanofibers via hydrogen bonding and hydrophobic interactions, forming large aggregates (State III). Bundled nanofibers restrict pHly-1 penetration into bacterial membranes, conferring low cytotoxicity against oral microbiota, gingival tissues, and mucosal surfaces at neutral pH (Figure 2) .^34^

### AMP coatings

Titanium and titanium alloys - the primary materials for oral implants - exhibit favorable biocompatibility and osseointegration capacity. AMP-based coatings on these surfaces effectively reduce device-associated bacterial infections.^35^ To inhibit bacterial colonization and peri-implantitis development, one preventive strategy involves immobilising natural AMPs onto titanium surfaces.^36^ Two immobilization methods exist: physical adsorption and covalent fixation. Research confirms that physically adsorbed AMP surfaces exhibit only transient, weak antibacterial effects compared to covalently immobilized surfaces. Covalently fixed AMPs ensure long-term surface retention without spontaneous dissociation while providing enhanced bioactivity.^37^ Covalent strategies include using silane, catechol, and phosphate groups to link AMPs to implant surfaces.^18^ For instance, researchers covalently anchored AMP GL13K to titanium via silane chemistry, creating a novel antibacterial coating resistant to mechanical, thermochemical, and enzymatic degradation while maintaining antimicrobial activity and cytocompatibility. Alternatively, I. Mutreja et al. developed a thiol-maleimide Michael addition-based approach, where cysteine-substituted GL13K (Cys-GL13K) undergoes chemoselective reaction with maleimide-functionalized titanium (eTi) surfaces, also forming effective antibacterial coatings.^38^ Another strategy involves coating implant surfaces - particularly transmucosal segments - with bioactive peptides. These coatings enable high-affinity binding to gingival epithelial cell receptors, promoting soft tissue seal formation where epithelial cells act as physical barriers; locally released AMPs inhibit bacteria and disrupt biofilm formation on titanium surfaces.^36^

## AMP antibiofilm mechanisms

### Inhibition of planktonic bacterial growth

AMPs exert antibacterial effects through cell wall-targeting or membrane-targeting mechanisms. Membrane-targeting mechanisms include transmembrane pore models ("barrel-stave model" and "toroidal-pore model") and non-pore models ("carpet model" or "aggregate model").

*Barrel-Stave Model:* Monomeric peptides initially accumulate on the cell surface, then undergo conformational changes and aggregation, forming barrel-shaped oligomers within the bacterial membrane. Aggregation facilitates AMP insertion into the hydrophobic membrane core while shielding hydrophilic regions from hydrophobic exposure. Hydrophobic chains interact with membrane acyl chains, aligning with the lipid bilayer core and inducing membrane destabilization. Hydrophilic segments form aqueous pores that expand with increasing peptide aggregation.^39^

*Toroidal-Pore Model:* AMPs remain largely inactive or exhibit low activity at low concentrations but transition to high activity at elevated concentrations. Hydrophilic domains bind lipid polar head groups while hydrophobic regions associate with nonpolar tails, reorienting from a position parallel to the phospholipid bilayer to one perpendicularly inserted into the lipid membrane.^40^ This displacement induces inward membrane curvature, generating 1-2 nm pores in the hydrophobic core due to the presence of AMP aggregates. Consequently, genetic material leakage and osmotic dysregulation lead to cell death.^41^

*Carpet Model:* In contrast to the barrel-stave and toroidal-pore models, AMPs initially interact with negatively charged moieties on the membrane surface and align parallel to it, covering the surface like a "carpet". Upon reaching a critical concentration threshold, AMPs act in a detergent-like manner, partially solubilising the lipid membrane and forming micelles. This leads to membrane disintegration, loss of integrity, and leakage of intracellular contents. Additionally, in an aggregation model, AMPs lack a specific orientation and embed into the cell membrane, forming micelle-like complexes with phospholipid molecules. These aggregates traverse the membrane, creating transient channels or pores that allow AMPs to cross the membrane and exert their antibacterial effects intracellularly.

**Fig. 3:**
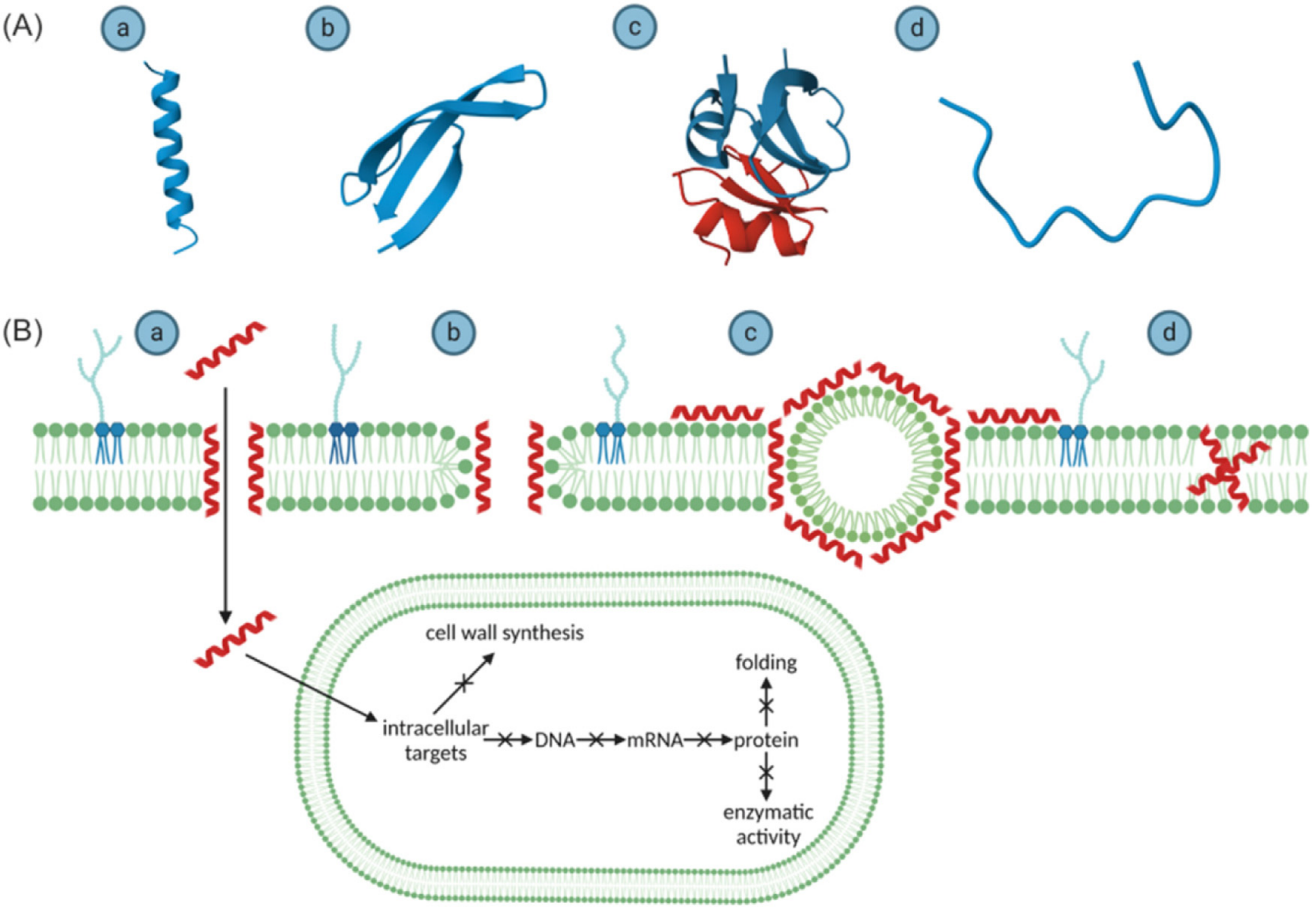
(A) α-helical peptides (B) β-sheet peptides (C) peptides containing both α-helical and β-sheet conformations (D) linear peptides (A) Four main models for membrane pore formation: (A) barrel-stave model (B) toroidal-pore model (C) carpet model (D) aggregation model. After penetrating the cell membrane, AMPs can interact with bacterial intracellular molecules, inhibiting DNA, RNA, and protein synthesis, protein folding, protein enzymatic activity, and the synthesis of cell wall components (B). Adapted with permission from Ref.^42^^,^^43^

Furthermore, AMPs can utilize intracellular targeting mechanisms. These involve translocation across the membrane into the cytoplasm, where they interfere with intracellular molecules and metabolic processes. Examples include inhibiting DNA, RNA, and protein synthesis, promoting the release of lytic enzymes to disrupt cellular structures, or inhibiting enzymes crucial for cell wall synthesis, ultimately leading to bacterial cell death (Figure 3).^42^^,^^43^

### Inhibiting the initial adhesion of bacterial cells to attachment surfaces, exerting antibiofilm activity

The adhesion of planktonic bacteria to object surfaces is key to initiating bacterial biofilm formation. Studies show that the minimum biofilm inhibitory concentration (MBIC) of LL-37 in saliva and gingival crevicular fluid is far lower than its minimum inhibitory concentration (MIC) for bacterial growth, indicating that its primary direct antibacterial effect is likely to prevent biofilm formation. This antibiofilm effect might result from short-term exposure to LL-37 stimulating bacterial twitching motility, which causes bacteria to wander on the surface, thereby reducing bacterial cell attachment instead of forming biofilms.^44^

### Modulating the bacterial biofilm quorum sensing (QS)

After free bacteria adhere to an attachment surface, they monitor changes in the surrounding environment by secreting QS signaling molecules, regulate the expression of their related genes, and secrete large amounts of extracellular polymeric substances (EPS) to fill the gaps between bacteria within the biofilm, providing mechanical stability for the bacterial biofilm. Therefore, the QS system is closely related to bacterial biofilm formation, the expression of pathogenicity-related virulence factors, and multiple drug resistance pathways. Gj-CATH2 exhibits strong bactericidal and antibiofilm effects against S. mutans. One of its mechanisms of action is inhibiting the expression of the QS systems (luxS and comD/E), leading to reduced EPS synthesis.^45^

### Inhibiting the bacterial stringent response

**Fig. 4:**
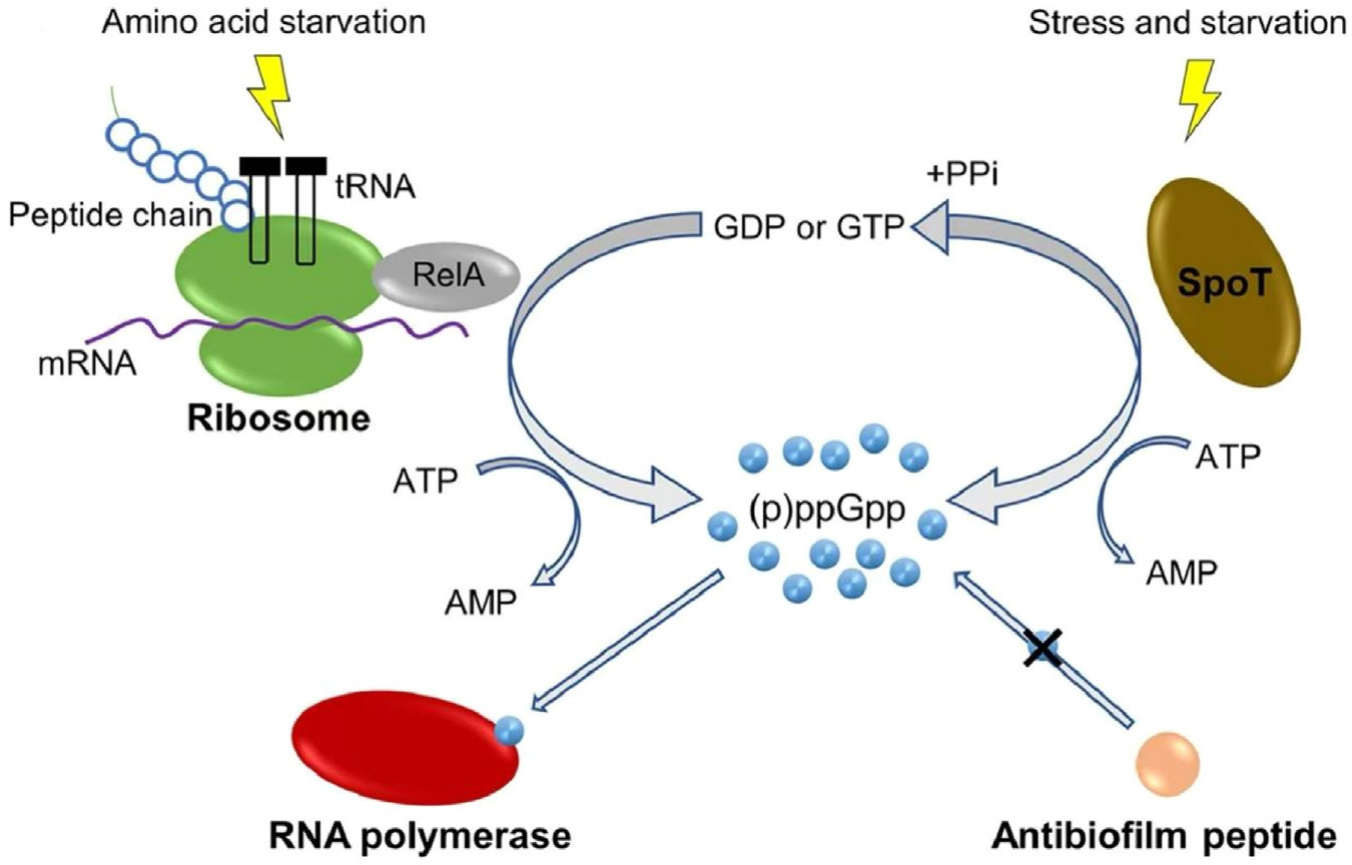
Antibiofilm peptides act by targeting a universal stress response in bacteria. ppGpp (produced in response to amino acid starvation or other stresses) signaling molecules bind to the RNA polymerase, which affects transcription. Rapid ppGpp accumulation results in a switch from cell growth to survival, and anti-biofilm peptides can block intracellular ppGpp accumulation. Adapted with permission from Ref.^48^

When bacteria suffer from nutrient starvation and environmental stress, the signaling molecule guanosine tetraphosphate/pentaphosphate [(p)ppGpp] is synthesized under the catalysis of the metabolic enzymes RelA/SpoT, thereby inducing bacterial cells to shut down the transcription of rRNA, tRNA, and ribosomal protein genes, halt the translation of multiple proteins, and strictly control most metabolic activities to enable bacterial survival.^46^ Therefore, any change in (p)ppGpp content severely affects bacterial biofilm formation and maintenance. Research has found that AMPs can exert antibiofilm activity by acting on the stringent stress response, which is universally present in both Gram-negative and Gram-positive bacteria. IDR1018, DJK-5, and DJK-6 can inhibit biofilm formation by blocking (p)ppGpp biosynthesis and promoting its degradation(Figure 4).^47^^,^^48^

### Downregulating the expression of binding protein transport genes responsible for biofilm formation, reducing biofilm formation

Nal-P-113 can inhibit P. gingivalis biofilm formation by downregulating genes encoding ABC transporters and ATP-binding proteins, such as PG0282 and PG1663.^49^

For pre-formed biofilms, AMPs can interfere with the bacterial membrane potential within the biofilm, disrupting bacterial membranes to degrade EPS. For instance, P1 can degrade EPS produced by S. mutans.^49^ However, at different biofilm stages, the same AMP can exert its biological activity in distinct ways. For example, G3 can inhibit bacterial adhesion in the initial stage by reducing surface charge, hydrophobicity, membrane integrity, and the transcription of adhesion-related genes. In subsequent stages, G3 interacts with extracellular DNA, disrupting the 3D structure of mature biofilms and dispersing them through the degradation of polysaccharides and the biofilm matrix.^50^

## Application of AMPs in oral biofilms

### Application of AMPs in cariogenic biofilms

S. mutans, as one of the primary pathogenic agents of dental caries,^51^ possesses strong acid-producing and acid-resistant capabilities, enabling it to grow competitively in acidic environments. It can also utilize sucrose as a substrate to synthesize glucan, also known as exopolysaccharides (EPS), via glucosyltransferases. The presence of EPS enhances the localized adhesion strength of S. mutans to tooth surfaces and promotes bacterial adhesion and aggregation. Finally, under continuous sucrose intake, it can continuously produce EPS in situ to expand the 3-dimensional matrix, providing a scaffold for the colonization and adhesion of other microorganisms, further leading to the formation of cariogenic biofilms.^52^ Over time, the sustained long-term decrease in biofilm pH leads to demineralization of tooth hard tissues and the development of dental caries.

De novo designed AMPs: Jiang et al. designed the cationic amphipathic α-helical GH12 based on the α-helical sequence (XXYY)n (where X denotes hydrophobic residues, Y hydrophilic residues, and n repeat count). They used the basic histidine (His, H) and the hydrophobic leucine (Leu, L) to form the main body of the amphipathic α-helical AMP, glycine (Gly, G) as the N-capping residue. To facilitate anchoring the peptide to the lipid bilayer surface, tryptophan (Trp, W) was positioned at the amphipathic interface between hydrophilic and hydrophobic faces. Finally, the C-terminus was amidated to synthesize GH12 (GLLWHLLHHLLH-NH_2_). This AMP effectively inhibits lactic acid production, EPS synthesis, pH decrease, and biofilm formation in human dental plaque-derived multi-species biofilms.^53^ Subsequently, other researchers suggested that the histidine-rich sequence of GH12 enables it to exhibit stronger antibiofilm effects against S. mutans biofilms at acidic pH.^54^ Therefore, leveraging the pH sensitivity of histidine, they engineered the pH-responsive LH12 (GLLHLHLLHLLH-NH_2_). This AMP inhibits cariogenesis through a dual-functional mechanism: targeting cariogenic pathogens in response to the acidified microenvironment, inhibiting the expression of multiple virulence genes and 2-component signal transduction systems, while simultaneously elevating the production of H_2_O_2_ commensal bacteria, thereby enhancing the ecological competitiveness of commensal bacteria.^55^

Template-based AMPs: Temporins, first discovered in amphibians, are among the naturally occurring shortest peptide families. To obtain derivative peptides with antibiofilm efficacy, researchers replaced histidine (His, H) at the GHa position with arginine (Arg, R) to engineer GHaR and GHa11R. Compared to GHa, both GHaR and GHa11R exhibited increases in charge, amphipathicity index (AI), and Boman index (BI). The 2 AMPs displayed selectively strong antimicrobial activity against oral pathogens, including S. mutans, S. sanguinis, and P. gingivalis. Furthermore, they also demonstrated antibiofilm activity against S. mutans by reducing surface hydrophobicity, interfering with initial adhesion, and inhibiting the production of virulence factors including lactic acid and EPS, while simultaneously reducing bacterial acidogenicity and aciduricity, and ultimately preventing dental caries development in vivo.^31^

### Application of AMPs in periodontal disease biofilms

Periodontitis is an inflammatory disease associated with microbial biofilm formation and gingival recession. The biofilm bacteria and their toxins disrupt gingival epithelial cells, triggering a cascade of inflammatory and immune processes. This leads to the destruction of gingival tissues and, ultimately, in susceptible patients, to alveolar bone loss and tooth loss due to periodontal disease. Over 500 distinct bacterial strains have been identified in dental plaque. Periodontitis-associated pathogens primarily include: A. actinomycetemcomitans, P. gingivalis, Actinomyces viscosus, P. intermedia, F. nucleatum, T. denticola, and T. forsythia.^56^ These bacteria can survive on tooth surfaces, within gingival epithelium, and in the oral environment, gradually forming structurally complex dental plaque biofilms.

Template-based AMPs: P-113 (AKRHHGYKRKFH-NH_2_) is a peptide derived from the salivary protein histatin 5. One research group synthesized the novel cationic Nal-P-113 by replacing histidine with β-naphthylalanine. This new AMP was demonstrated to possess resistance to proteolytic degradation and high-salt conditions. It not only retains its antimicrobial activity under physiological conditions but also kills planktonic bacteria at low concentrations. Furthermore, at higher concentrations, it kills bacteria within biofilms without harming rat oral mucosa.^57^ Additionally, another research group designed and optimized the novel DP7 (VQWRIRVAVIRK-NH_2_) based on amino acid prediction methods. This AMP has been confirmed to exhibit bactericidal effects against *P. gingivalis*. It also inhibits biofilm formation by disrupting bacterial membranes and suppressing the expression of several virulence factors.^58^ Lactoferrin chimera (LFchimera) is a heterodimeric peptide constructed by linking 2 antimicrobial domains of the mammalian defense protein lactoferrin. It has been reported that LFchimera can inhibit the formation of in vitro multi-species biofilms derived from subgingival plaque samples of periodontitis patients harbouring A. actinomycetemcomitans. Moreover, it reduces the viability of multi-species bacteria within biofilms more effectively than CHX or MH.^59^

### Application of AMPs in Root Canal Infection Biofilms

Periapical biofilm refers to a bacterial biofilm located on the root cementum surface beyond the apex or on the surface of overextended root canal filling materials. Predominant microbial species frequently identified within these biofilms include Actinomyces, E. faecalis, Propionibacterium, Fusobacterium, and Pseudomonas.^60^ Effective disinfection of the root canal system presents a significant challenge, attributable to its inherent complexity and variability, coupled with the multispecies nature of the biofilm.

Natural AMPs: Nisin, a natural AMP produced by L. lactis,^61^ has demonstrated efficacy in inhibiting E. faecalis biofilms when evaluated as an experimental intracanal medicament. Lysozyme, a naturally occurring antimicrobial enzyme present in mucosal secretions of animals and humans, also functions as a cationic antimicrobial peptide (CAMP). Planktonic E. faecalis exhibits intrinsic resistance to the bactericidal activity of lysozyme, mediated by a transmembrane metalloprotease named Eep.^62^ However, this Eep-mediated resistance is absent when E. faecalis resides within a biofilm. This observation confirms that the CAMP activity of lysozyme, rather than its muramidase activity, is responsible for the biofilm-specific killing of E. faecalis.^63^ Im-4, an immune peptide induced in Drosophila upon activation of the Toll innate immune system during fungal defense, has also proven particularly effective in reducing biofilm formation.^64^

Template-based AMPs: buCaTHL4B, a cathelicidin-4 homolog characterized by a high tryptophan content, has been established as an effective AMP against dental plaque biofilms.^65^ Consequently, studies have investigated the antibiofilm effects of buCaTHL4B and Im-4 against biofilms formed by E. faecalis and F. nucleatum. At a concentration of 10μg/mL, both buCaTHL4B and Im-4 exhibited significantly higher bactericidal activity compared to 1% NaOCl. Notably, Im-4 demonstrated superior efficacy over buCaTHL4B in preventing biofilm formation.^66^ Additionally, HBD3-C15, a synthetic peptide comprising the 15 C-terminal amino acids of human β-defensin-3, has also shown inhibitory activity against 24-hour E. faecalis biofilms formed on human dentin blocks.^67^

De novo designed AMPs: DJK-5 is a synthetic AMP with the sequence VQWRAI-RVRVIR. Its antimicrobial potency is closely linked to its amphipathic molecular structure, which confers resistance to degradation by both bacterial and host proteases, resulting in enhanced activity compared to natural AMPs. DJK-5 penetrates cell membranes and targets intracellular nucleotides, specifically degrading guanosine tetraphosphate (ppGpp), thereby suppressing biofilm accumulation.^47^ Research demonstrates that DJK-5 is effective against both single-species (E. faecalis) and oral multispecies biofilms cultivated on hydroxyapatite and dentin substrates.^67^ M33D, another AMP, is a tetra-branched peptide synthesized using D-amino acids; its branched architecture provides resistance to degradation by plasma, serum, and bacterial proteases. By replacing the isoleucine residue in M33D with leucine, researchers synthesized the analog M33i/l. Evaluated as root canal disinfectants, both M33D and M33i/l act rapidly against bacteria and effectively disrupt biofilms. Furthermore, M33i/l generates fewer chemical byproducts and offers potential cost advantages.^68^

### Application of AMPs in peri-implantitis biofilms

Peri-implantitis can develop from peri-implant mucositis. Its clinical manifestations include soft tissue inflammation and alveolar bone loss.^69^ Dental plaque biofilm is the primary etiological factor for peri-implant mucositis,^70^ analogous to gingivitis around natural teeth. With appropriate plaque control, this condition is reversible. However, inadequate control of the plaque biofilm at this stage can lead to progression to peri-implantitis, even in the presence of established osseointegration between the implant and alveolar bone.^71^ While initial biofilm formation by Streptococcus represents a significant etiological factor,^72^ substantial evidence indicates that the peri-implantitis microbiota constitutes a heterogeneous polymicrobial infection. This community is associated not only with taxa linked to periodontitis but also includes opportunistic pathogens such as P. aeruginosa, S. aureus, and C. albicans.^73^

Template-based AMPs: Zhang et al. designed a novel short peptide, VR-12 (VRLWVRVRRWRR-NH_2_), based on the scaffold of IDR-1018. VR-12 significantly inhibits key peri-implantitis pathogens in both planktonic and biofilm states.^74^ The design strategy involved substituting tryptophan for the fourth isoleucine residue, capitalising on the enhanced membrane penetration capability of tryptophan-rich AMPs. To introduce cationic charge and hydrogen-bonding potential, arginine was substituted for the sixth alanine and ninth isoleucine residues. These modifications facilitate interaction with anionic components in bacterial membranes, thereby augmenting antimicrobial activity.^74^

AMP coatings: Wisdom et al. engineered a bifunctional peptide comprising 3 distinct domains: an implant-anchoring domain provided by a titanium-binding peptide (TiBP), an antimicrobial domain provided by an AMP, and a spacer domain. They incorporated a rigid, extended amino acid spacer ("GSGGG") between the TiBP domain and the antimicrobial domains derived from 2 different AMPs: GL13K (GKIIKLKASLKLL) or AMPA (KWKLWKKIEKWGQGIGAVLKWLTTW). These bifunctional peptides were shown to selectively bind to titanium/titanium alloy implant surfaces, forming an antimicrobial peptide coating within just 2 minutes. Binding efficiency approached 100% even in the presence of bacterial contamination and serum proteins. Critically, this bifunctional peptide coating can be applied to new implants and also re-applied to previously placed implants to control bacterial colonization and mitigate biofilm formation.^18^

**Fig. 5:**
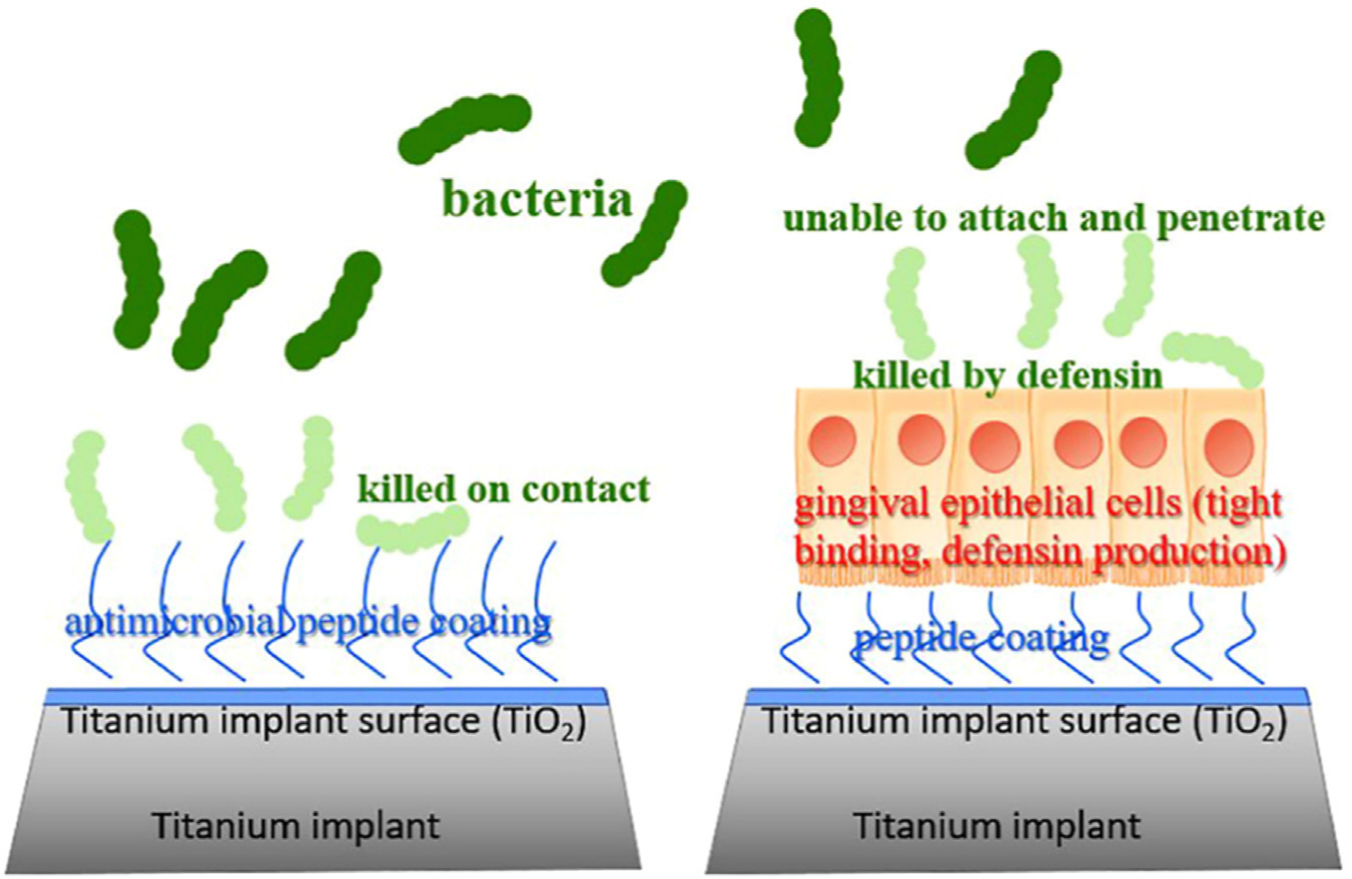
Two preventive strategies for inhibiting bacterial colonization and peri-implantitis development on peptide-coated titanium surfaces: (A) based on surface-immobilised AMPs that kill bacteria directly on contact. (B) based on bioactive coating of implant-surfaces by peptides which bind to the surface receptors of gingival epithelial cells with high affinity. The gingival epithelial cells function as a physical barrier inhibiting bacterial colonization on titanium surface. Furthermore, the AMPs produced and released by epithelial cells also interfere with biofilm formation. Adapted with permission from Ref.^36^

Another strategy involves coating implant surfaces - particularly transmucosal segments - with bioactive peptides. These coatings enable high-affinity binding to gingival epithelial cell receptors, promoting soft tissue seal formation where epithelial cells act as physical barriers; locally released AMPs inhibit bacteria and disrupt biofilm formation on titanium surfaces (Figure 5).^36^

### Application of AMPs in candida biofilms

Candida species constitute 30%-60% of the fungal component within the natural human oral microbiota, typically persisting as commensal organisms without causing symptomatic disease in healthy individuals. These fungi primarily exist in a planktonic state, colonising diverse oral surfaces ranging from the vulnerable mucosal epithelium to hard dental structures and prosthetic appliances.^75^ However, immunocompromised individuals experience increased oral Candida colonization, which can progress to oral candidiasis. In this pathogenic state, Candida demonstrates the capacity to form biofilms and hyphae, and produces virulence factors including hydrolytic enzymes and candidalysin. Despite mucosal immune activation, the synergistic increase in microbial load and virulence - initiated by the production of hyphae-associated toxins from colonising C. albicans cells - collectively drives infection.^75^^,^^76^ Furthermore, within the oral environment, C. albicans frequently associates with bacterial biofilms. Substantial research has established that interactions between C. albicans and various oral bacteria contribute significantly to the pathogenesis of biofilm-associated oral diseases, encompassing dental caries, oral candidiasis, endodontic diseases, periodontitis, implant-related infections, and oral cancer.^77^

*De Novo Design:* Zou et al. synthesised 5 novel α-helical antimicrobial peptides (ACPs 1-5) by integrating structural features from natural antifungal and nonantifungal peptides. They comprehensively evaluated these ACPs for antimicrobial efficacy, antibiofilm properties, activity against drug-resistant fungi, and underlying mechanisms. Notably, the ACPs induced apoptosis in biofilm-forming C. albicans cells that exhibited minimal or no susceptibility to fluconazole. This finding highlights the potent fungicidal activity of these peptides against fluconazole-resistant strains and suggests a low propensity for resistance development. The ACPs also demonstrated significant efficacy in suppressing both hyphal formation and biofilm development.^78^

*Template-Based Design:* Researchers identified and modified a novel AMP, designated Octominin, derived from the cDNA sequence of defensin 3 in the little octopus. Initial studies confirmed Octominin's antimicrobial activity against C. albicans and its anti-inflammatory properties.^79^ Subsequently, a truncated 18-amino acid variant, Octominin II, was developed. Comprehensive assessment of its antifungal profile - including determination of minimum inhibitory/bactericidal concentrations (MIC/MFC), analysis of ultrastructural damage, membrane permeability assays, transcriptional effects, and antibiofilm activity - revealed a multifaceted mechanism of action. Octominin II inhibits C. albicans through physical disruption of the cell membrane, increased plasma membrane permeability, induction of high levels of reactive oxygen species (ROS), and binding to intracellular DNA and RNA. Crucially, it also exhibits potent antibiofilm activity, effectively inhibiting formation and eradicating established C. albicans-derived biofilms.^80^

## Limitations, concluding remarks, and future perspectives

As discussed above, peptide DJK-5 outperformed conventional mouthrinses and CHX in killing oral multispecies biofilms with a constant, high proportion of dead bacteria.^47^ Although natural products such as propolis show potential in dentistry due to their antimicrobial and anti-inflammatory properties supported by laboratory and clinical studies, their translation into reliable therapies is hindered by significant limitations, including complex composition, inconsistent efficacy, and lack of standardization.^81^ Furthemore, other "natural" rinses such as saline, baking soda, and cocout oil show conflicting evidence regarding clinical efficacy.^82^ In this context, AMPs represent a promising therapeutic strategy, though many current in vitro studies on AMPs may not fully replicate in vivo or clinical conditions. They offer potential for targeted delivery against pathogens, modulation of host immune responses, "balancing" of oral microbiome, and possibly reducing the burden of oral hygiene maintenance. Therefore, future efforts should focus on translating AMP-based strategies into dental applications, aiming to enhance their safety, efficacy and specificity while reducing production costs to facilitate their transition from costly experimental agents to viable therapeutics.

## Conflict of interest

None disclosed.
